# A novel combination of bortezomib, lenalidomide, and clarithromycin produced stringent complete response in refractory multiple myeloma complicated with diabetes mellitus – clinical significance and possible mechanisms: a case report

**DOI:** 10.1186/s13256-017-1550-6

**Published:** 2018-02-18

**Authors:** Nobuo Takemori, Goro Imai, Kazuo Hoshino, Akishi Ooi, Masaru Kojima

**Affiliations:** 1 Division of Hematology, Department of Internal Medicine, Imai Hospital, Tanaka-cho 100, Ashikaga, Tochigi 326-0822 Japan; 2 Department of Internal Medicine, Imai Hospital, Tanaka-cho 100, Ashikaga, Tochigi 326-0822 Japan; 3 Department of Surgery, Imai Hospital, Tanaka-cho 100, Ashikaga, Tochigi 326-0822 Japan; 4 Department of Molecular and Cellular Pathology, School of Medicine, Kanazawa University, 13-1 Takara-machi, Kanazawa, Ishikawa 920-8640 Japan; 5 Department of Diagnostic Pathology, Dokkyo Medical University School of Medicine, Shimotsuga-gun, Mibu-machi, Tochigi 321-0293 Japan

**Keywords:** Combination chemotherapy, Clarithromycin, Diabetes mellitus, Refractory multiple myeloma, Stringent complete response

## Abstract

**Background:**

In general, dexamethasone is a required component drug in various combination chemotherapies for treating multiple myeloma, and its efficacy has been widely recognized. However, administration of dexamethasone is known to cause various adverse effects including hyperglycemia which requires insulin therapy. During the course of treatment, we developed a novel effective dexamethasone-free combination regimen and evaluated it for its effect in multiple myeloma.

**Case presentation:**

We report a case of 68-year-old Japanese woman with refractory advanced Bence-Jones-λ type multiple myeloma associated with diabetes mellitus. Various combination regimens were carried out, but the response to some regimens was insufficient or others containing dexamethasone, although effective, were inappropriate to continue due to aggravation of diabetes mellitus. Thus, we developed a dexamethasone-free, short dosing-period regimen consisting of bortezomib, lenalidomide, and clarithromycin. This regimen was found to be highly effective and succeeded in achieving stringent complete response.

**Conclusions:**

The successful dexamethasone-free regimen clearly shows that dexamethasone is not a requisite component in treating multiple myeloma, and it can be substituted with clarithromycin. This regimen is particularly useful for treating patients with multiple myeloma associated with diabetes mellitus.

## Background

Lenalidomide (Len; Revlimid®) has been used in combination with dexamethasone (Dex) for treating relapsed/refractory multiple myeloma (MM) [1], newly diagnosed MM [2, 3], and transplant-ineligible MM [4]. Subsequently, bortezomib (Bor; Velcade®), the first-in-class proteasome inhibitor of the 26S proteasome, was approved in combination with Dex for treating newly diagnosed MM prior to autologous stem cell transplantation [5]. Recently, both drugs have been widely used for treating MM. In general, high-dose/low-dose Dex is a requisite component in various combination chemotherapies for treating MM, and its efficacy has been recognized. However, administration of Dex is known to cause various adverse effects including hyperglycemia leading to secondary diabetes mellitus (DM), DM-aggravation, DM-nephropathy, DM-retinopathy, reactivation of hepatitis virus B leading to fulminant hepatitis, increased susceptibility to infections, Cushing syndrome, psychic disturbance like delirium, systemic osteoporosis, cataract, and so on. Sometimes, clinicians have to treat patients with MM who also have DM. In such cases, regimens that include Dex induce severe hyperglycemia which requires insulin therapy. We treated a patient with refractory Bence-Jones (BJ)-λ type MM associated with DM, and succeeded in achieving stringent complete response (sCR) with a Dex-free, short dosing-period (sdp) regimen in which clarithromycin (CAM) was combined with Bor and Len.

## Case presentation

Our patient was a 68-year-old Japanese woman (48 kg/151 cm) with a 7-year history of DM. She had a past history of receiving a radical mastectomy for right mammary cancer in the department of surgery of our hospital at the age of 65. Her postoperative course was uneventful. After the operation, she received chemotherapies (trastuzumab, paclitaxel, and tegafur-uracil) until the end of December 2014, and later she was followed without treatment on an out-patient basis. In March 2015, she showed progressive unexplained anemia. On 4 June 2015, she was admitted to the surgical department of our hospital at the age of 68 because of anemia, abdominal pain, paralytic ileus, generalized neuralgia-like pains, and bilateral shoulder joint swellings and pains. On 13 June 2015, she was referred and admitted to the hematologic division for further evaluation.

Physical examinations on admission were as follows. Her temperature was 36.5 °C, her pulse was 88, and her blood pressure was 130 systolic and 85 diastolic. Her right mamma was completely removed with a remaining operation scar. She appeared chronically ill and could not turn over due to generalized neuralgia-like pains and severe right shoulder pains. Her skin showed pallor and palpebral conjunctivae were anemic. Her abdomen was distended, tympanitic, and diffusely tender without peristalsis, indicating paralytic ileus. Superficial lymph nodes were impalpable.

Laboratory data on admission were as follows. A complete blood cell count showed moderate anemia (red blood cell count, 2.31 × 10^12^/L; hemoglobin 7.4 g/dL), slightly increased leukocytes (white blood cell count, 9.5 × 10^9^/L with 67.5% neutrophils, 17.5% lymphocytes, and 13% monocytes, 2% eosinophils) and normal platelet count (301 × 10^9^/L). Elevated levels of serum free light chain-λ (FLC-λ; more than 3200 mg/L; normal range (nr.), 4.44~26.18 mg/L), β_2_-microglobulin (BMG; 6.5 mg/L; nr. 0.9~1.9 mg/L), soluble-interleukin (IL)-2 receptors (1300 U/mL; nr. 124~466 U/mL), ferritin (955.6 ng/mL; nr. 3.6~114 pg/mL), CRP (1.74 mg/dL; nr. 0~0.26 mg/dL), D-dimer (8.69 μg/mL; nr. < 0.72 μg/mL), and fibrin degradation product (FDP; 20.1 μg/mL; nr. < 5 μg/mL), N-terminal pro-brain natriuretic peptide (NT-proBNP; 191 pg/mL; nr. ≤ 125 pg/mL), CA19-9 (42 U/mL; nr. ≤ 37 U/mL), sialyl-Le^x^-i (SLX; 42.8 U/mL; nr. ≤ 38 U/mL), glycated hemoglobin (HbA1c; 6.7%; nr. 4.54~6.25%) and uric acid (7.2 mg/dL; nr. 2.5~7.0 mg/dL), and depressed levels of immunoglobulin (Ig) G (341 mg/dL; nr. 870~1700 mg/dL), IgA (15 mg/dL; nr. 110~410 mg/dL), IgM (10 mg/dL; nr. 46~260 mg/dL), IgD (<1.0 mg/dL; nr. ≤ 12.6 mg/dL), total protein (6.4 g/dL; nr. 6.7~8.3 g/dL), albumin (3.8 g/dL; nr. 3.9~4.9 g/dL), zinc turbidity test (ZTT; 1 KU; nr. 4~12 KU), serum Fe (50 μg/dL; nr. 54~181 μg/dL) and total cholesterol (106 mg/dL; nr. 130~219 mg/dL) were observed. Values of carcinoembryonic antigen (CEA), aspartate aminotransferase (AST), alanine aminotransferase (ALT), lactate dehydrogenase (LDH), creatine phosphokinase (CPK), amylase, blood urea nitrogen (BUN), creatinine, and electrolytes were within normal range. Titers of rheumatoid arthritis particle agglutination, anti-nuclear antibody, anti-microsome antibody, and anti-thyroglobulin antibody were within normal range. Her urine showed proteinuria (7.4 g/day). Lambda-type BJ protein was detected in her urine by immunofixation electrophoresis. Bone scintiscan using ^99m^Tc-hydroxymethylene diphosphonate (HMDP) revealed multiple uptakes of radioisotope in generalized bones, particularly in vertebrae and shoulder joints. Chest and abdominal computed tomography scans revealed massive soft tissue masses around bilateral shoulder joints suggestive of amyloid light-chain (AL) deposition (shoulder-pad sign), paralytic ileus, small multiple mesenteric lymph nodes, small amount of ascites, slight right hydronephrosis, and slight splenomegaly. Skull X-ray films showed sporadic punched out lesions.

**Fig. 1:**
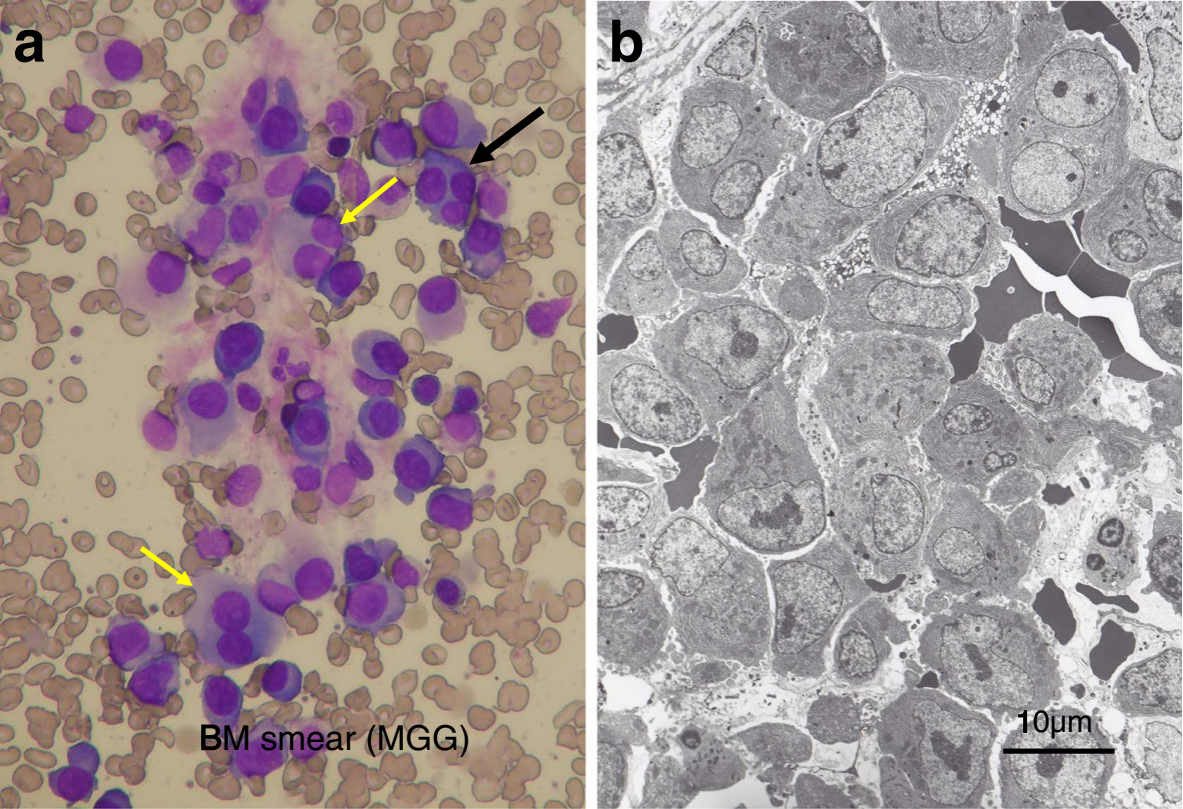
Bone marrow obtained before the initiation of treatment. **a** Bone marrow smear. Bone marrow is infiltrated by immature myeloma cells showing basophilic cytoplasm and fine nuclear chromatin networks with occasional nucleoli. Binuclear (*yellow arrows*) and trinuclear (*black arrow*) myeloma cells, suggesting hyperdiploidies, are seen (under × 40 magnification objective). **b** Electron micrograph of the bone marrow. Bone marrow is compactly occupied by immature myeloma cells with remarkable nucleoli, fine chromatin networks, and abundant rough endoplasmic reticula

An iliac bone marrow (BM) puncture performed on 16 June 2015 showed that her BM was infiltrated by atypical medium-sized and large-sized myeloma cells with basophilic cytoplasm and fine chromatin networks with occasional nucleoli. Binuclear and trinuclear myeloma cells were frequently seen (Fig. 1). Myeloma cells accounted for 70.8% of total nucleated cells on smear preparation. On immunohistochemical examination, they were exclusively positive for light chain λ.

**Fig. 2:**
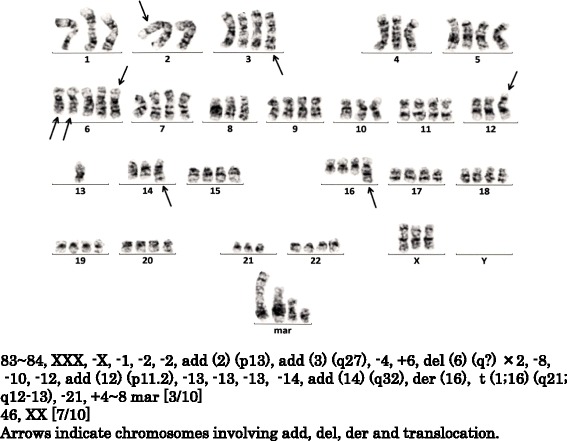
Chromosome analysis showing complex hyperdiploidies. Hyperdiploidies were seen in three out of ten cells (3/10). Seven out of ten cells showed a normal karyotype (7/10). Similar hyperdiploidies remained until achieving stringent complete response

A chromosomal analysis of the BM cells revealed complex hyperdiploidies (Fig. 2). Thus, a diagnosis of BJ-λ type MM (Durie and Salmon, stage IIIA; International Staging System, stage III; Southwest Oncology Group, stage III) was made.

In the hematologic division of our hospital, most of the patients with MM are vulnerable older people with severe complications. Thus, the dose of Len is minimized to 10 mg/day in those patients to avoid the adverse effects, such as leukopenia, thrombocytopenia, toxicoderma, deep venous thrombosis, neuropathy, a high fever associated with elevated CRP (that is, transient inflammatory reaction), and so on. In fact, 10 mg of Len was found to be effective enough for treating MM with reduced adverse effects, and allowing for safe repetitions of treatments [6]. Moreover, doses of Bor and Dex are also reduced to improve tolerability and to optimize efficacy as suggested by Palumbo *et al*. [7].

**Fig. 3:**
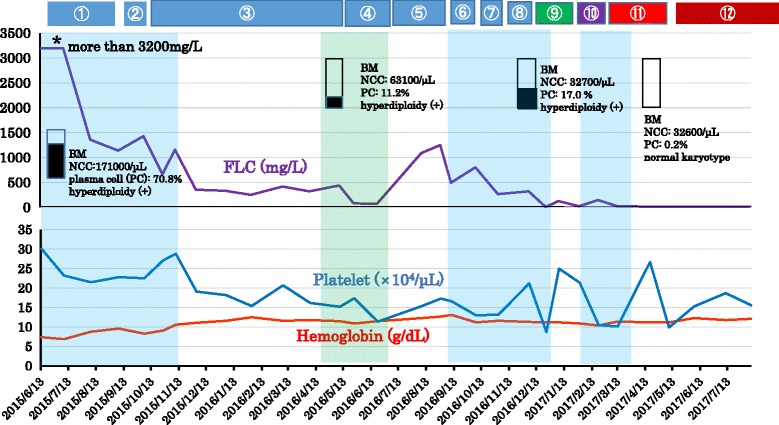
Whole clinical course of the patient. Regimen ➀ (VAD) was fairly effective for reducing free light chain. Levels of free light chain-λ were depressed to 1140 mg/L from more than 3200 mg/L. Afterwards, various combination chemotherapies were carried out; however, the responses to some regimens were insufficient or other regimens, including Dex, although effective, were inappropriate to continue the treatments due to aggravation of diabetes mellitus. Stringent complete response was achieved by initiation of regimen ⑪ (sdpBR^Md^-CAM^800^). Levels of hemoglobin gradually elevated to a normal range, and platelet levels fluctuated within normal range. *Faint blue areas* indicate hospitalization. ① VAD; ② R^Md^D; ③ R^Md^D-CAM^400^; ④ sdp BD; ⑤ B^Red^D; ⑥ PD; ⑦ sdp BD; ⑧ VAD; ⑨ sdp BD-CAM^800^; ⑩ sdp B-CAM^800^; ⑪ sdp BR^Md^-CAM^800^; ⑫ sdpB^Red^R^Md^-CAM^800^. *BD* Bor-Dex, *BM* bone marrow, *BR* Bor-Revlimid (lenalidomide), *CAM* clarithromycin, *FLC* free light chain, *Md* minimized dose, *NCC* nucleated cell count, *PC* plasma cell, *PD* pomalidomide-Dex, *red* reduced dose, *sdp* short dosing-period, *VAD* vincristine, adriamycin, and Dex

**Fig. 4:**
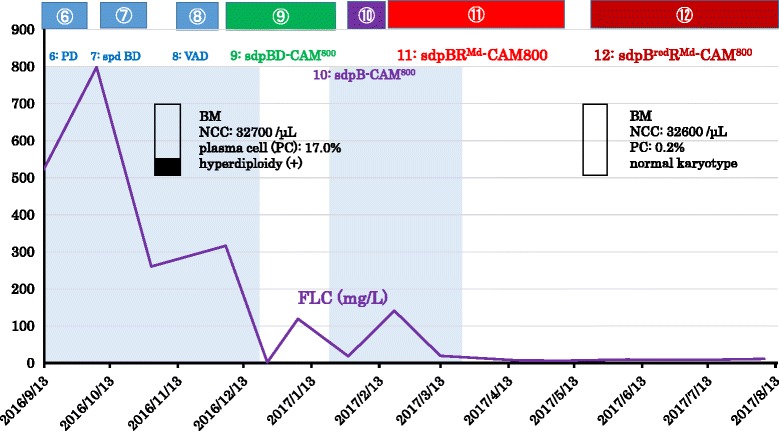
Detailed clinical course after September 2016. After the initiation of regimen ⑪ (sdpBR^Md^-CAM^800^),  stringent complete response was obtained and is still maintained with regimen ⑫ (sdpB^Red^R^Md^-CAM^800^) as of October 2017. Myeloma cells disappeared from BM and hyperdiploidies also disappeared. Regimen ⑨ (sdpBD-CAM^800^) was also effective and the level of FLC-λ was depressed to normal range, but this treatment could not be continued due to aggravation of diabetes mellitus. *BD* Bor-Dex, *BM* bone marrow, *BR* Bor-Revlimid (lenalidomide), *CAM* clarithromycin, *FLC* free light chain, *Md* minimized dose, *NCC* nucleated cell count, *PC* plasma cell, *PD* pomalidomide-Dex, *Red* reduced dose, *sdp* short dosing-period, *VAD* vincristine, adriamycin, and Dex

The whole clinical course (Fig. 3), the detailed clinical course (Fig. 4), and regimens used during the whole clinical course are listed in Table 1. At first, three cycles of ① vincristine, adriamycin, and Dex (VAD) therapy were carried out. This treatment was fairly effective; levels of FLC-λ and BMG were depressed to 1140 μg/L from more than 3200 μg/L and to 2.2 mg/L from 6.5 mg/L, respectively. Bilateral shoulder joint pains and paralytic ileus were rapidly improved. She was released from confinement to bed and able to walk 3 weeks after starting the first cycle of VAD therapy. Although this regimen proved to be effective for relieving her symptoms, levels of FLC-λ still remained high (1140 mg/L). As shown in Table 1 and Fig. 3, regimens ①~⑫ were consecutively carried out. In general, the regimens including Dex caused severe hyperglycemia which required insulin therapy, and most of them, except regimen ③ in which CAM (400 mg/day for 3 weeks) was combined with Len and Dex, could not be efficiently repeated due to DM aggravation or inefficacy of the regimens. Of all the regimens, regimen ⑨ (short dosing-period [sdp] Bor-Dex-CAM^800^), and the regimen ⑪/⑫ (sdpB^(Reduced dose [Rd]^ R^Minimized dose^ ^[Md]-^CAM^800^) proved to be very effective. Levels of FLC-λ were drastically depressed to a normal range by regimens ⑨ and ⑪ (Fig. 4). However, the former could not be repeated because of the aggravation of DM, leading to deterioration of the general condition of our patient. On the other hand, regimen ⑪ could be safely repeated without fearing DM aggravation; the levels of FLC-λ were brought down to a normal range (12.9 mg/L) with normal κ/λ ratio (1.024) after four cycles of this regimen (Table 1). Myeloma cells disappeared from her BM (Figs. 3 and 4) and BJ proteins disappeared from her serum and urine. Two color flow cytometric analysis of the BM showed absence of clonal plasma cells (PCs). In addition, levels of serum IgG, IgA, and IgM elevated to normal levels. Thus, our patient was proved to be in sCR according to the updated criteria of International Myeloma Working Group (IMWG) [8]. In May 2017, urticaria started to appear after subcutaneous (sc) injections of Bor. Thus, the administration of Bor was reduced to once a week from twice a week (regimen ⑫: sdpB^Red^R^Md^-CAM^800^) and intravenous administration of hydrocortisone 100 mg, prior to each Bor injection, was started to prevent urticaria. Regimen ⑫ was safely repeated without fearing DM aggravation as well as urticaria. Levels of FLC-λ remained within normal range, and our patient is in good condition as of November 2017.

**Table 1:** Brief summary of regimens used during the whole clinical course

Regimen	Cycles	Date of treatments	FLC-λ(mg/L)/BMG(mg/L)	Evaluation
① VAD	3	Jun. 19~Sep.5/2015	≥ 3200→1140/6.5→2.2	Effective
②R^Md^D	1	Sep.14 ~Oct. 4/2015	1140→1430/2.2→2.5	Ineffective
③ R^Md^D-CAM^400^	7	Oct. 12~May 15/2016	1430→439/2.5→1.5	Effective
④ sdp BD	2	May 16~Jun. 29/2016	439→72.9/1.5→1.3	Effective
⑤ B^Red^D	2	Jul. 11~Sep. 6/2016	72.9→496/1.3→1.5	Ineffective
⑥ PD	1	Sep. 13~Oct. 3/2016	496→805/1.5→1.9	Ineffective
⑦ sdpBD	1	Oct. 17~Oct. 30/2016	805→267/1.9→1.6	Fairly effective
⑧ VAD	1	Nov. 14~Dec. 6/2016	267→323/1.6→1.4	Ineffective
⑨sdpBD-CAM^800^	2	Dec. 12~Jan. 23/2017	323→24.9/1.4→1.1	Very effective
⑩sdpB-CAM^800^	1	Feb. 4~Feb. 26/2017	24.9→148/1.1→2.1	Ineffective
⑪ sdpBR^Md^-cam^800^	4	Feb. 27~May 8/2017	148→12.9/2.8→2.1	Very effective
⑫ sdpB^Red^R^Md^-CAM^800^	6	May 23~Oct. 31/2017	12.9→14.2/2.1→1.4	Effective/stable

*A (ADM)* adriamycin, *B* bortezomib, *BMG* ß2-microglobulin, *CAM* clarithromycin, *D (Dex)* dexamethasone, *FLC* free light chain, *Md* minimized-dose, *P (Pom)* pomalidomide, *R* Revlimid (Len: lenalidomide), *Red* reduced dose, *sc* subcutaneous, *sdp* short dosing-period, *V* vincristine, *wk* week

① VAD: V 0.4 mg/body·ADM 9 mg/m^2^, continuous intravenous infusion, on days 1–4; Dex 40 mg/body, on days1–4, 9–12, 17–20, every 4-wk cycle

②R^Md^D: Len 10 mg/day for 3 wks; Dex 20 mg/day, twice/week for 3 wks, every 4-wk cycle

③R^Md^D-CAM^400^: R^Md^D combined with CAM 400 mg/day, 200 mg twice/day for 3 wks, every 4-wk cycle

④ sdpBD: Bor 1.3 mg/m^2^, sc, twice/wk for 2 wks; Dex 20 mg/day twice/wk for 2 wks, every 4-wk cycle

⑤ B^Red^D: Bor 1.3 mg/m^2^, sc, once/wk for 4 wks; Dex 20 mg/day, twice/wk for 4 wks, every 6-wk cycle

⑥ PD: Pom 4 mg/day for 3 wks; Dex 20 mg/day twice/wk for 3 wks, every 4-wks cycle

⑦ sdpBD ⑧VAD

⑨ sdpBD-CAM^800^: sdpBD combined with CAM 800 mg/day, 400 mg twice/day for 2 wks, every 4-wk cycle

⑩sdpB-CAM^800^: Bor 1.3 mg/m^2^, sc, twice/wk for 2 wk; CAM 800 mg/day, 400 mg twice/day for 2 wks, every 4-wk cycle

⑪sdpBR^Md^-CAM^800^: Bor 1.3 mg/m^2^, sc, twice/wk for 2 wks; Len 10 mg/day for 2 wks; CAM 800 mg/day, 400 mg twice/day for 2 wks, every 4-wk cycle

⑫sdpB^Red^R^Md^-CAM^800^: Bor 1.3 mg/m^2^, sc, once/wk for 2 wks; Len 10 mg/day for 2 wks; CAM 800 mg/day, 400 mg twice/day for 2 wks, every 4-wk cycle

## Discussion

It is important to elucidate the mechanisms whereby sCR is achieved by the combination regimen consisting of Len, Bor and CAM. Bor, the 26S proteasome inhibitor, has been widely used for treating MM [9]. Increasing lines of evidence indicate that inhibition of the 26S proteasome by Bor, that is, blocking of the ubiquitin proteasome system by Bor, leads to the accumulation of unfolded or misfolded protein in the endoplasmic reticulum (ER) in myeloma cells; this results in ER stress followed by a coordinated cellular response known as unfolded protein response (UPR) [10–13]. UPR is known to induce activation of the chaperone protein GRP78 (Bip) to maintain ER integrity and upregulates transcription factor CHOP (the C/EBP homologous protein, also designated as GADD153) to mediate cell death when ER stress is beyond the tolerance of the cell adaptation [10–13]. In the clinical field, Bor is now widely used in combination regimens such as VMP (Bor, melphalan, and prednisolone), VTD (Bor, thalidomide, and Dex), PVD (Pomalidomide, Bor, and Dex), or BRD (Bor, Len, and Dex) in refractory/relapsed MM or newly diagnosed MM. Although regimens involving Bor and Dex have contributed to substantial improvement of MM, the treatment of patients with MM associated with DM is still a troublesome issue, and therapeutic improvements are required.

As for CAM, many investigators have reported its immunosuppressive or immunomodulatory effects in patients with cancers or tumor-bearing animals. In brief, CAM is known to decrease the production of IL-1, IL-2, IL-5, IL-6, IL-8, IL-10, tumor necrosis factor (TNF)-α, transforming growth factor (TGF)-α, TGF-β, and matrix metalloproteinase 9, and increase the production of IL-4, IL-12, and interferon–γ [6, 14, 15]. In addition, CAM is known to induce apoptosis through Fas-Fas ligand pathway [16].

Recently, the high efficacy of the chemotherapeutic regimen combining CAM (Biaxin®) with Len and Dex (BiRD regimen) in treating MM has been documented [17, 18]. Concerning the role of CAM in BiRD therapy, the steroid-enhancing/sparing effect is suggested [6, 14, 17, 19]; however, the exact mechanisms of action of CAM remain uncertain. Nakamura *et al*. [20] reported that CAM attenuates autophagy and induces cell growth inhibition in MM cells; more exactly, the treatment with CAM attenuates autophagy by blocking the late phase of the autophagic process, probably after the fusion of autophagosomes with lysosomes in myeloma cells.

Although CAM by itself exhibits no cytotoxicity [6, 12, 21], simultaneous inhibition of the ubiquitin-proteasome system by Bor and autophagy-lysosome system by CAM will synergistically lead to activate UPR, resulting in enhanced MM cell apoptosis [12, 22].

Since MM is characterized by uncontrolled cell growth of monoclonal antibody-producing neoplastic PCs, large quantities of unfolded or misfolded Ig production itself triggers ER stress [22]. This may mean that MM is a specific neoplasm susceptible to proteasome and autophagy inhibitors. In support of this proposal, Obeng *et al*. [11] reported that MM cells have a lower threshold for proteasome inhibitor-induced UPR induction and ER stress-induced apoptosis.

In BiRD regimen, CAM at a dose of 1000 mg/day, 500 mg twice a day, was recommended [17, 18]. In the present case, we applied a relatively high dose of CAM (800 mg/day, 400 mg twice a day, for 2 weeks) in regimens ⑨~⑫, and succeeded in achieving and maintaining sCR by the regimen ⑪/⑫ without any severe adverse effects.

Len has generally been used in combination with Dex for treating refractory and relapsed MM [1], newly diagnosed MM [2, 3], and transplant-ineligible MM [4]. The precise cellular targets and the exact mechanisms of action of Len remain unclear. However, recent studies revealed that Len’s multiple effects include: (1) *immune modulation* (CD4+ and CD8+ T cell co-stimulation, regulatory T cell suppression, Th1 cytokine production, NK and NKT cell activation, and antibody-dependent cellular cytotoxicity), (2) *interference with tumor micro-environment interactions* (anti-angiogenesis, anti-inflammatory properties, downregulation of adhesion molecules, and anti-osteoclastogenic properties) and (3) *direct anti-tumor effects* (inhibition of cyclin-dependent kinase, induced expression of tumor suppressor genes such as Egr-1, *Egr-2*, and *SPARC* genes, downregulation of NF-κB, and inhibition of caspase 3, 8, and 9) [23]. Len is known to suppress TNF-α, IL-1β, IL-6, IL-12, TGF-β, macrophage inhibitory protein-α, granulocyte-macrophage colony-stimulating factor, insulin-like growth factor-1, basic FGF, RANKL, and vascular endothelial growth factor (VEGF), and to increase IL-2, IL-10, and interferon-γ. Inhibition of VEGF by Len may alter the BM microvasculature, thereby making the microenvironment less hospitable for MM cell growth [6, 22–25]. Recently, Krönke *et al*. [26] demonstrated that Len causes selective ubiquitination and degradation of two lymphoid transcription factors – Ikaros family zinc finger proteins (IKZF) 1 and 3 (IKZF1, Ikaros; and IKZF3, Aiolos) – by the CRBN-CRL4 ubiquitin ligase. It is known that IKZF1 and IKZF3 are essential B cell transcription factors. A study in mice demonstrated that IKZF3 is required for the generation of PCs [27]. In this connection, Lu *et al*. [28] reported that Len-bound cereblon acquires the ability to target IKZF1 and IKZF3 for proteasomal degradation in MM cells. They analyzed myeloma cell lines and demonstrated that loss of IKZF1 and IKZF3 is necessary and sufficient for Len’s therapeutic effect. In the present case, we demonstrated that regimen ⑪/⑫ (sdpBR^Md^-CAM^800^ or sdpB^Red^R^Md^-CAM^800^) is highly effective for treating MM. Possibly, Len’s diverse apoptosis-inducing effects might further enhance cytotoxic effects of Bor and CAM.

In general, Dex is considered to be a requisite for treating MM, and it is included in most of the regimens for MM. However, in the present case, sCR was easily achieved by a Dex-free regimen (sdpBR^Md^-CAM^800^ or sdpB^Red^R^Md^-CAM^800^).  This seems to indicate that Dex is not necessarily requisite in treating MM. Nevertheless, the effect of regimen ⑨ (sdpBD-CAM^800^), which includes Dex, was equal to regimen ⑪ (sdpBR^Md^-CAM^800^), but the former was discontinued because of severe hyperglycemia which required insulin therapy. In contrast, the effect of Len-omitted regimen ⑩ (sdp B-CAM^800^) was ineffective. This means that the combination of three components, namely, Bor, Len, and CAM are requisite for treating MM.

It is well known that CAM is the potent CYP3A4 inhibitor and Bor is metabolized by CYP3A4. Therefore, concomitant administration of Bor and CAM may lead to intracellular elevation of Bor concentrations [12]. One may ask whether the enhanced cytotoxicity achieved by regimens including Bor and CAM is simply due to the pharmaco-interaction between Bor and CAM via CYP3A4. We cannot completely exclude this possibility.

## Conclusions

In the present study, we clearly demonstrated that sdpBR^Md^-CAM^800^ or sdpB^Red^R^Md^-CAM^800 ^regimen is highly effective for treating refractory MM. This regimen has outstanding merits as follows. (1) The regimen is Dex-free, therefore there is no risk of developing steroid-induced DM/DM aggravation or DM-related complications. (2) The regimen is based on a dose-reduction schedule, the adverse effects of Bor and Len are attenuated, and its medical cost is less expensive. (3) The regimen is based on a short-term schedule; it is less risky in developing various adverse effects and gives patients enough time to recover, enabling clinicians to repeat it safely. (4) The regimen contains CAM which might play an important role in enhancing synergistically the effects of Bor and Len. The sdpB^(Red)^R^Md^-CAM^800^  regimen is a promising combination therapy for patients with MM particularly associated with DM.

## Abbreviations

BD: Bortezomib-dexamethasone
BJ: Bence-Jones
BM: Bone marrow
BMG: β_2_ microglobulin
Bor: Bortezomib
CAM: Clarithromycin
Dex: Dexamethasone
DM: Diabetes mellitus
ER: Endoplasmic reticulum
FLC: Free light chain
Ig: Immunoglobulin
IKZF: Ikaros family zinc finger proteins
IL: Interleukin
Len: Lenalidomide
MM: Multiple myeloma
nr.: Normal range
PC: Plasma cell
sCR: Stringent complete response
sdp: Short dosing-period
TGF: Transforming growth factor
TNF: Tumor necrosis factor
UPR: Unfolded protein response
VAD: Vincristine, adriamycin, and dexamethasone
VEGF: vascular endothelial growth factor
